# Peony (*Paeonia lactiflora*) leaf performance of two cultivars in response to ozone exposure

**DOI:** 10.3389/fpls.2025.1607998

**Published:** 2025-07-11

**Authors:** Zaisheng Shao, Liquan Jing, Li Zha, Xiaoyan Lu, Wenhui Huo, Kai Yang, Hengfeng Zhang

**Affiliations:** ^1^ Jiangsu Agri-Animal Husbandry Vocational College, Taizhou, Jiangsu, China; ^2^ Jiangsu Key Laboratory of Crop Genetics and Physiology/Jiangsu Key Laboratory of Crop Cultivation and Physiology/Jiangsu Co-Innovation Center for Modern Production Technology of Grain Crops, Agricultural College of Yangzhou University, Yangzhou, China

**Keywords:** peony (*Paeonia lactiflora*), air pollution, abiotic stress, leaf photosynthetic capacity, antioxidant system

## Abstract

**Introduction:**

High surface ozone (O_3_) concentration presents a significant threat to the growth and development of plants. This research aimed to evaluate the impact of O_3_ on peony (*Paeonia lactiflora*), focusing on the physiological mechanisms involved, mainly as peony is widely grown in O_3_ polluted regions such as Sichuan, Jiangsu, and Shandong.

**Methods:**

Two cultivars of peony Dafugui (DFG) and Heihaibotao (HHBT) were exposed to either non-filtered air (NF, ambient O_3_ concentration) or elevated O_3_ (NF60, NF + 60 ppb) for fifty days (April 11 to May 30) in open-top chambers. Key physiological parameters, including gas exchange, pigment levels, leaf mass per area (LMA), stomatal structure, lipid oxidation, and the antioxidant defense system, were measured across three replicate chambers.

**Results:**

The exposure to NF60 resulted in significant reductions in light-saturated photosynthesis rate (*A_sat_*), stomatal conductance (*g_s_*), and electron transfer rate (ETR), but no changes were observed in intercellular CO2 concentration (Ci). Increased O_3_ levels accelerated leaf senescence, as indicated by higher malondialdehyde (MDA) and hydrogen peroxide (H_2_O_2_) levels, along with a decline in chlorophyll content. Ozone-induced led to a reduction in LMA, but the stomatal area and density were not significantly affected. The total ascorbate (AsA) content was decreased but total antioxidant capacity (TAC), phenolics, and antioxidant enzyme activity showed an increasing trend due to O_3_. Statistical analysis using ANOVA revealed no significant differences in the responses of leaf indices between the two peony cultivars to O_3_ stress, as indicated by the absence of significant interaction effects between O_3_ treatment and cultivar.

**Discussion:**

The above results indicate that under O_3_ stress, peony leaves exhibit chlorosis and aggravated membrane lipid peroxidation, leading to a decline in Asat and inhibited leaf growth. The findings underscore the necessity of cultivating and promoting ozone-tolerant peony cultivars in heavily O_3_ polluted areas to improve the production efficiency and quality of the peony.

## 1 Introduction

Surface ozone (O_3_) is predominantly produced through photochemical reactions involving primary pollutants, such as nitrogen oxides (NO_X_) and volatile organic compounds (Monks et al., 2015; Li K, et al., 2018). The increasing levels of NO_X_, driven by industrial development and the rapid expansion of vehicle ownership, have led to higher concentrations of O_3_ in the atmosphere (Jiang et al., 2012; Feng et al., 2015). Environmental monitoring data reveal that between 2013 and 2019, the daily maximum 8-hour average O_3_ concentration in the surface layers of many Chinese cities consistently exceeded 50 ppb, with peak O_3_ levels increasing at an annual rate of 3 ppb (Lu et al., 2020). In some areas, such as Jiangsu province, the maximum hourly average concentration has exceeded 100 ppb (Hu et al., 2023; Shao et al., 2023). These increased concentrations of surface O_3_ present significant threats to human health (Sicard et al., 2021; Marco et al., 2022), agricultural production (Feng et al., 2022; Ramya et al., 2023), and biodiversity (Ainsworth, 2017).

The herbaceous peony (*Paeonia lactiflora*) is a well-known traditional flower in China, valued for its ornamental, medicinal, and health-promoting properties. It has emerged as a commercially significant cut flower in both domestic and international markets due to its substantial economic benefits (Ning et al., 2015; Qi et al., 2020). China has become one of the world’s major producers of cut peonies (Yang et al., 2020), with production concentrated in the Beijing, Sichuan, Jiangsu, and Shandong provinces (Du et al., 2018). Extensive research has been conducted on the effects of various abiotic stressors on peony growth, including heavy metals, saline-alkali conditions, drought, waterlogging, temperature extremes, and insufficient light. Lu et al. (2022) reviewed these stressors and their impacts on peony morphology, internal physiological processes, and secondary metabolites. These findings provided valuable insights into the mechanisms by which peonies adapt to stress and improve their tolerance. However, unlike other environmental stressors, O_3_ pollution has seasonal variations that make certain crops, including peony, particularly vulnerable during their critical growth phases. Moreover, China’s major peony-producing regions also experience frequent high O_3_ concentrations (Feng et al., 2022), particularly during the crucial growth and flowering period in May, which coincides with regional O_3_ pollution peaks (Wang et al., 2001; Li et al., 2007; Xu et al., 2008a; Ding et al., 2013; Wang et al., 2017). Despite numerous studies highlighting the harmful effects of O_3_ on crops (Feng and Kobayashi, 2009; Peng et al., 2019; Shao et al., 2020; Shang et al., 2024) and woody species (Li et al., 2016; Shang et al., 2018; Dai et al., 2017), minimal research has been conducted on the impacts of O_3_ on horticultural plants (Mills et al., 2007; Zhang et al., 2015), particularly on peony, to date.

Furthermore, leaves serve as the primary interface for material and energy exchange between the surface atmosphere and the terrestrial biosphere, and they are also the main organs responsible for sensing O_3_ stress responses (Krupa and Manning, 1988). Ozone predominantly enters plants through the stomata. Prolonged exposure to O_3_ induces visible leaf injury symptoms in sensitive plants species (Mills et al., 2011; Feng et al., 2014), activates various defense mechanisms, and subsequently leads to progressive chlorophyll degradation, accompanied by a reduction in photosynthetic efficiency (Wittig et al., 2009). These physiological disruptions interfere with carbon assimilation and the distribution of essential mineral nutrients (Shang et al., 2018), ultimately causing growth retardation and premature aging (Matyssek and Sandermann, 2003). Therefore, the plant’s resilience to environmental stress, its ecological fitness, its capacity for carbon sequestration are all compromised (Feng et al., 2022). Significant variability in O_3_ tolerance has been observed both among crops (Mills et al., 2007; Paoletti et al., 2009) and within species (Biswas et al., 2008; Krupa et al., 1998; Maggs and Ashmore, 1998; Shi et al., 2009).

In crops, susceptibility to O_3_ is typically measured by reductions in yield and biomass. Whereas, the evaluation of O_3_ sensitivity in woody and ornamental plants often involves monitoring leaf traits such as specific leaf weight, photosynthetic performance, malondialdehyde (MDA) levels, and antioxidant enzyme activity. Furthermore, high-concentration, short-term O_3_ exposure often induces oxidative damage in the form of leaf necrosis, accompanied by increased antioxidant metabolism, as a result of programmed cell death. However, the plant’s antioxidant response to chronic exposure to lower O_3_ concentrations is less well understood and remains a subject of debate. Some studies suggest that chronic O_3_ stress leads to increased antioxidant activity, as observed in soybeans (Gillespie et al., 2011), while others have observed a reduction in antioxidant pools and key enzymes in wheat leaf tissues under elevated O_3_ concentrations (Feng et al., 2010; Wang et al., 2014a). No studies have examined how O_3_ affects peony’s antioxidant system or photosynthetic efficiency. Therefore, a systematic study on the effects of O_3_ elevation on peony leaf performance is essential to determine its sensitivity to O_3_ stress. In this study, O_3_ fumigation experiments were conducted under two distinct concentrations, non-filtered ambient O_3_ concentration (NF) and NF supplemented with an additional 60 ppb of O_3_ (NF60), to evaluate the physiological responses of two widely cultivated cultivars of *Paeonia lactiflora*. The primary objective was to elucidate how O_3_ exposure influences leaf morphological traits, photosynthetic physiology, and oxidative stress responses in the two peony cultivars and to provide an initial evaluation of cultivar sensitivity to O_3_ exposure, offering foundational insights for optimizing peony cultivation practices in O_3_-polluted regions.

## 2 Materials and methods

### 2.1 Plant material and cultivation

Using Dafugui (DFG) and Heihaibotao (HHBT) as experimental materials, both cultivars were transplanted by division in 2023. This experiment used pot cultivation management with a soil mixture comprising a 1:1:1 ratio of soil, peat, and coarse sand. The cultivation pot is a plastic container with a lower base diameter of 18 cm, an upper opening diameter of 25 cm, and a height of 27.5 cm. The soil in the pot with the following properties: soil organic matter 14.1 ± 0.8%, total nitrogen (N) 1.48 ± 0.01 g kg^-1^, available phosphorus (P) 17.2 ± 4.8 mg kg^-1^, available potassium (K) 40.4 ± 2.1 mg kg^-1^, and pH6.6. The fertilizer and water management for the potted plants were consistent with standard field-level practices. In brief, throughout the trial period, water the plants when the top 2–3 cm of soil becomes dry, while avoiding waterlogging. In late April, apply 20 g of monopotassium phosphate per pot and then water thoroughly.

### 2.2 Fumigation treatment

The O_3_ fumigation experiment was initiated in April 2024 at the Yangzhou Green Agriculture Research and Demonstration Base. The experimental design utilized open-top chambers (OTCs) of regular octagonal geometry (4.8 m diameter, 2.3 m height, and a 3 m diameter top opening). Two O_3_ treatments were applied: ambient O_3_ concentration (NF) and ambient air supplemented with 60 ppb O_3_ (NF60), with each treatment replicated across three OTCs under comparable environmental conditions (temperature, humidity, illumination). Ozone was produced from high-purity oxygen using an electrical discharge O_3_ generator (HY003, Jinan Chuangcheng Technology Co., Ltd.) and delivered to the chambers by blowers (CX-125, Shanghai Quanfeng Industrial Co., Ltd.). Canopy-level O_3_ concentrations was continuously monitored in real-time (at 1-minute intervals) using Thermo Scientific Model 49i analyzers. Ozone delivery was fine-tuned *via* mass flow controllers to match set-point concentrations. Ozone levels were regulated by adjusting oxygen flow rates through mass flow controllers based on deviations between measured and target concentrations. Three uniformly growing *Paeonia lactiflora* pots per cultivar were randomly placed in each OTC starting April 11. Fumigation was carried out for 10 hours daily (08:00–18:00) over 50 days ending May 30. The average O_3_ concentrations during this period were 43.7 ± 1.1 ppb (NF) and 98.2 ± 1.4 ppb (NF60).

### 2.3 Parameter measurements

Leaf samples were collected from three plants per cultivar in each chamber. The uppermost three leaves from a single stem of each plant were pooled to create a composite sample. Leaves were punched, immediately frozen in liquid nitrogen, and milled to a fine powder using a miller (MM400, Retsch, Arzberg, Germany). These samples were analyzed for chlorophyll content, MDA, total ascorbate (AsA), total antioxidant capacity (TAC), hydrogen peroxide accumulation (H_2_O_2_), phenolic, and antioxidant enzyme activities.

Chlorophyll content in leaf samples was determined following the method described by Wang et al. (2014b), with minor modifications. Approximately 30 mg of fresh leaf tissue was extracted using 1 mL of 95% ethanol and incubated for 4 hours at 30°C in a shaker incubator (QYC 2102C, FuMa Experimental Equipment Co., Ltd., Shanghai, China). After centrifugation, the absorbance of the supernatant was measured at 645, 663, and 470 nm using a microplate reader (Lambda 35, PerkinElmer, Norwalk, CT, USA). Based on these values and according to established protocols (Wang et al., 2014b), the concentrations of chlorophyll a, chlorophyll b, total chlorophyll (a + b), and carotenoids were calculated.

Leaf mass per area (LMA): The top three leaves from each of the three single stems of each plant were first collected. The leaf length and surface area were measured using Image J software. The leaves were then combined and placed into paper bags, dried at 105°C for 30 minutes, followed by 72 hours at 80°C, to obtain dry weight. The leaf mass per area of each peony cultivar was estimated using the area and dry weight of the upper three leave.

To evaluate stomatal characteristics, three plants from each cultivar were selected, and the uppermost fully expanded leaves were sampled. After gently removing the dust, a layer of transparent nail polish was applied to a 1 cm × 1 cm area of the abaxial leaf surface. Once dried, the film was lifted with adhesive transparent tape and mounted onto a microscope slide with the impression side facing up. Observations were performed using a Leica DM 2500 microscope, and stomatal density was calculated by counting stomata within a defined area (Laza et al., 2010). The lengths and widths of 10 randomly selected stomata were measured per sample, and the stomatal area was calculated as length × width.

Gas exchange parameters were recorded using a portable photosynthesis system (LI-6800, LI-COR Inc., Lincoln, NE, USA). Measurements included the light-saturated net photosynthetic rate (*A_sat_*), stomatal conductance (*g*_s_), intercellular CO_2_ concentration (*C_i_*), and electron transfer rate (*ETR*) of three fully expanded upper leaves from a single plant, randomly selected in each chamber. All measurements were conducted between 8:30 and 11:30 a.m. on sunny days, with saturation-level photosynthetic active radiation (1200 μmol m^-2^·s^-1^), 400 ppm of CO_2_, leaf temperatures of 28°C, and relative humidity readings of 50-60%.

The concentration of MDA in leaf tissues was measured using microplate-based spectrophotometric analysis as described by Wang et al. (2014b). The TAC was assessed using the ferric reducing antioxidant power (FRAP) assay, which reflects the collective action of non-enzymatic antioxidants and provides an indicative measure of the leaf’s resistance to oxidative stress (Benzie and Strain, 1996). The AsA concentration was determined spectrophotometrically by measuring absorbance at 265 nm after ascorbate oxidase addition, using an extinction coefficient of 14–14 mM^-1^ cm^-1^ (Luwe and Heber, 1995). Phenolic content in the leaf supernatant was analyzed using the Folin–Ciocalteu reagent following the protocol of Gillespie and Ainsworth (2007), with results expressed in gallic acid equivalents. Leaf hydrogen peroxide (H_2_O_2_) levels were determined using the method described by Mukherjee and Choudhuri (1983).

The activities of the antioxidant enzymes, including superoxide dismutase (SOD), peroxidase (POD), and hydrogen peroxidase(CAT), were analyzed using specific commercial kits from Jiancheng Bioengineering Institute (kit codes A123-1-1, A084-1, A007-1, and A001-4), with spectrophotometric detection. Leaf samples (~20 mg) were homogenized in 2 mL of pH 7.8 extraction buffer and centrifuged at 10,000 × g at 4 °C for 15 minutes. The enzyme activities were determined in the clarified supernatant, following the manufacturer’s protocol.

### 2.4 Statistical analysis

The statistical analysis was performed using SAS 9.2 software (SAS Institute Inc., Cary, NC). A mixed-effects model was used, following the approach described by Frei et al. (2011), where the fixed effects included O_3_, cultivar, and their interactions, and the random effect was the chambers. This mixed model did not consider multiple plants within one treatment chamber as replicates on the treatment level. All the data were presented as the mean ± standard error from three chamber replicates.

## 3 Results

### 3.1 Leaf gas exchange parameters

As presented in **Table 1**, exposure to NF60 significantly reduced the *A_sat_* and *g_s_* of peony leaves by an average of 23.0% and 25.6%, respectively. The reduction in DFG leaves was greater than that in HHBT, although no significant interaction was detected between O_3_ treatment and cultivar. A 12.7% significant reduction in *Ci* was observed in DFG leaves under NF60, while no significant changes were observed in HHBT, as indicated by the significant interaction between O_3_ exposure and cultivar. Moreover, NF60 exposure resulted in a significant 16.1% decrease in *ETR*, with similar reductions observed in both cultivars.

**Table 1:** Leaf photosynthesis parameters (light-saturated photosynthesis rate (*A_sat_*), stomatal conductance (*g_s_*), intercellular CO_2_ concentration (*C_i_*) and electron transfer rate (*ETR*)) of two cultivars of peony seedlings growing in nonfiltered ambient air (NF) and NF with a targeted O_3_ addition of 60 ppb (NF60).

Parameters	O_3_ treatment	DFG	HHBT	O_3_	Cultivar	O_3_ × Cultivar
*A*_sat_ (umol m^−2^ s^−1^)	NF	15.2 ± 0.60	12.51 ± 0.58	**0.007**	0.051	0.491
	NF60	11.4 ± 1.42	9.94 ± 0.75			
	%	-25.4	-20.6			
*g_s_* (mmol m^−2^ s^−1^)	NF	0.16 ± 0.01	0.14 ± 0.01	0.065	0.708	0.079
	NF60	0.11 ± 0.03	0.12 ± 0.02			
	%	-32.9	-18.3			
*C_i_* (umol mol^-1^)	NF	228.3 ± 8.8	236.6 ± 7.5	0.467	0.148	**0.037**
	NF60	199.3 ± 17.7	241.8 ± 12.1			
	%	-12.7	2.2			
*ETR* (umol m^−2^ s^−1^)	NF	161.7 ± 8.1	143.7 ± 14.2	**0.021**	0.113	0.698
	NF60	133.8 ± 6.5	122.2 ± 4.3			
	%	-17.3	-15.0			

Data are shown as Mean ± standard error (n = 3).

Statistically significant effects (*P* < 0.05) are marked in bold.

### 3.2 Leaf pigments

As shown in **Table 2**, the NF60 exposure resulted in an average 12.7% decrease in chlorophyll a+b content in peony leaves compared to NF (*P* < 0.1), with reductions of 7.1% in DFG and 18.4% in HHBT. The contents of chlorophyll a and b were also significantly decreased by 13.7% and 9.9%, respectively, under NF60. The reduction in chlorophyll content was less marked in DFG than in HHBT. However, no significant interaction was observed between O_3_ exposure and cultivar (**Table 2**).

**Table 2:** Leaf chlorophyll a+b, chlorophyll a and chlorophyll b of two cultivars of peony seedlings growing in nonfiltered ambient air (NF) and NF with a targeted O_3_ addition of 60 ppb (NF60).

Cultivar	O_3_ treatment	Chlorophyll a+b (mg g^-1^ FW)	Chlorophyll a (mg g^-1^ FW)	Chlorophyll b (mg g^-1^ FW)
DFG	NF	1.30 ± 0.08	0.97 ± 0.06	0.33 ± 0.02
	NF60	1.21 ± 0.12	0.90 ± 0.09	0.31 ± 0.03
	%	-7.1	-7.9	-4.6
HHBT	NF	1.02 ± 0.05	0.76 ± 0.03	0.26 ± 0.01
	NF60	0.83 ± 0.08	0.62 ± 0.06	0.22 ± 0.02
	%	-18.4	-19.4	-15.2
O_3_		0.087	0.067	0.185
Cultivar		**0.003**	**0.003**	**0.005**
O_3_ × Cultivar		0.513	0.508	0.530

Data are shown as Means ± standard error (n = 3).

FW, fresh weight

Statistically significant effects (*P* < 0.05) are marked in bold.

### 3.3 Leaf mass per area and stomata structure

As presented in **Table 3**, the dry weight per unit area of herbaceous peony leaves was determined based on leaf area and corresponding dry weight. Compared to NF, NF60 resulted in a 12.8% reduction in dry weight per unit area on average (*P* = 0.094), with DFG and HHBT showing reductions of 12.1% and 13.4%, respectively. Analysis using ANOVA indicated no significant effects of cultivar or the interaction between cultivar and O_3_ treatment on this parameter.

**Table 3:** Leaf mass per area (LMA), stomatal area and density of two cultivars of peony seedlings growing in nonfiltered ambient air (NF) and NF with a targeted O_3_ addition of 60 ppb (NF60).

Cultivar	O_3_ treatment	LMA (g m^-2^ DW)	Stomatal area (×10^-2^ mm^2^)	Stomatal density (mm^-2^)
DFG	NF	54.6 ± 3.39	14.0 ± 0.44	260.1 ± 15.4
	NF60	47.9 ± 4.67	13.6 ± 0.09	289.8 ± 10.7
	%	-12.1	-2.4	11.4
HHBT	NF	57.0 ± 3.98	14.4 ± 0.35	247.5 ± 13.7
	NF60	49.4 ± 2.78	14.3 ± 0.41	256.8 ± 20.3
	%	-13.4	-0.4	3.8
O_3_		0.094	0.398	0.225
Cultivar		0.608	0.403	0.164
O_3_ × Cultivar		0.895	0.534	0.506

Data are shown as Mean ± standard error (n = 3).

DW, dry weight.

### 3.4 Leaf stomata structure

The effect of O_3_ stress on leaf stomatal structure was shown in **Table 3**. Evaluation of stomatal structure revealed no significant differences in stomatal density between the cultivars; however, HHBT had significantly larger stomata than DFG. Neither O_3_ treatment nor its interaction with the cultivar significantly affected stomatal size or density (**Table 3**).

### 3.5 Leaf lipid peroxidation and antioxidant system

As shown in **Table 4**, O_3_ exposure resulted in significant increases in MDA (6.9%) and H_2_O_2_ (8.4%) contents in peony leaves, while carotenoid content decreased by 10.0%. No significant effects were observed on AsA, TAC, or phenolic contents. Under NF60 treatment, TAC content in HHBT leaves increased by 13.3%, whereas DFG showed a slight decrease, highlighting a significant interaction between O_3_ treatment and cultivar.

**Table 4:** Leaf lipid peroxidation and antioxidant system of two cultivars of peony seedlings growing in nonfiltered ambient air (NF) and NF with a targeted O_3_ addition of 60 ppb (NF60).

Parameters	O_3_ treatment	DFG	HHBT	O_3_	Cultivar	O_3_ × Cultivar
MDA (nmol g^-1^ FW)	NF	36.7 ± 1.10	36.3 ± 1.25	**0.042**	0.818	1.000
	NF60	39.2 ± 1.82	38.8 ± 2.50			
	%	6.8	6.9			
Carotenoid (mg g^-1^ FW)	NF	0.39 ± 0.02	0.31 ± 0.01	**0.085**	**0.002**	0.447
	NF60	0.37 ± 0.03	0.27 ± 0.01			
	%	-5.2	-14.8			
AsA (umol g^-1^ FW)	NF	1.73 ± 0.02	1.68 ± 0.05	0.459	0.159	0.830
	NF60	1.71 ± 0.03	1.65 ± 0.04			
	%	-1.2	-2.2			
TAC (umol g^-1^ FW)	NF	258.5 ± 12.3	239.1 ± 10.5	0.499	0.913	**0.041**
	NF60	256.3 ± 35.1	270.9 ± 16.2			
	%	-0.8	13.3			
H_2_O_2_ (mmol g^-1^ FW)	NF	4.70 ± 0.23	4.63 ± 0.24	**0.038**	0.909	0.801
	NF60	4.96 ± 0.83	5.15 ± 0.45			
	%	5.5	11.2			
Phenolics (umol g^-1^ FW)	NF	106.5 ± 6.2	113.1 ± 3.6	0.218	0.116	0.404
	NF60	109.9 ± 10.8	129.1 ± 18.4			
	%	3.4	14.1			
SOD (U g^-1^ FW)	NF	680.1 ± 34.3	679.5 ± 14.9	0.514	0.909	0.927
	NF60	701.6 ± 37.0	695.8 ± 19.8			
	%	3.2	2.4			
POD (U g^-1^ FW)	NF	66.4 ± 16.5	43.7 ± 5.0	0.073	**0.020**	**0.016**
	NF60	68.3 ± 8.7	53.1 ± 10.0			
	%	2.9	21.7			
CAT (U g^-1^ FW)	NF	104.7 ± 6.9	96.9 ± 10.5	0.635	**0.092**	0.635
	NF60	110.0 ± 9.1	96.9 ± 9.4			
	%	5.0	0.0			

Data are shown as Mean ± standard error (n = 3).

MDA, malondialdehyde; AsA, reduced ascorbic acid; TAC, total antioxidant capacity; H_2_O_2_, hydrogen peroxide accumulation; SOD, superoxide dismutase enzyme; POD, peroxidase enzyme; CAT, hydrogen peroxidase enzyme, FW, fresh weight.

Statistically significant effects (*P* < 0.05) are marked in bold.

Moreover, the activities of SOD and CAT enzymes were not significantly altered by O_3_ exposure, but POD activity increased by 12.3% (**Table 4**), this increase was greater in HHBT (21.7%) than in DFG (2.9%). The statistical analysis revealed that the O_3_-cultivar interaction significantly influenced POD activity alone.

## 4 Discussion

The Herbaceous peony (*Paeonia lactiflora Pall*.), renowned for its majestic beauty, is often compared to its tree (*Paeonia suffruticosa* Andr.) and is regarded as the “monarch of herbaceous flowers”. Its large, vibrant, and fragrant flowers have made it a popular choice for urban landscaping, garden cultivation, potted displays, and the premium cut flower market (Holloway and Buchholz, 2013). However, during the introduction and cultivation of the peony, the plant faces numerous abiotic stresses, including fluctuations in temperature, light exposure, water availability, saline-alkali conditions, and heavy metal contamination (Haak et al., 2017). While moderate stress can stimulate growth, excessive stress inhibits development. It causes morphological abnormalities in roots, stems, and leaves, altering internal organic compounds, inorganic ions, and enzymatic activities, which may ultimately lead to plant mortality (Zhang et al., 2018). Among the various abiotic stresses, elevated ground-level O_3_ concentrations have emerged as a key stress factor for plant health (Ashmore, 2005). Despite this, research on the impact of O_3_ stress in peony remains limited.

The leaf photosynthesis is an essential physiological process that sustains plant life. There are few reports on the effects of O_3_ stress on ornamental plants. Qin et al. (2020) conducted a study on O_3_ stress in *Cleome* sp*inosa*, but unfortunately, the study did not measure the response of leaf photosynthetic parameters. The impact of O_3_ stress on leaf photosynthesis has been the subject of substantial research, revealing that O_3_ exposure significantly reduces photosynthetic rates in various crops and tree species. Studies on wheat (Feng and Kobayashi, 2009; Xu et al., 2025), rice (Ainsworth, 2008; Shang et al., 2024), maize (Yendrek et al., 2017), and trees (Li et al., 2016; Shang et al., 2019) demonstrate that O_3_ impairs photosynthetic capacity. The present study showed that NF60 treatment resulted in a 23.0% significant decrease in the photosynthetic rate in peony leaves compared to NF (**Table 1**), with similar reductions in both cultivars, indicating significant O_3_-induced damage. Moreover, the reduction in photosynthetic rate in peony leaves under NF60 treatment was lower compared to the reductions observed in rice (Ainsworth, 2008; Shao et al., 2023), wheat (Feng et al., 2016), maize (Yendrek et al., 2017), and soybeans (Morgan et al., 2003). The observed decrease in photosynthesis can be attributed to both stomatal and non-stomatal limitations (Dai et al., 2017; Shang et al., 2024). Furthermore, under NF60, stomatal conductance was significantly reduced, with DFG showing a greater reduction than HHBT. However, O_3_ exposure also reduced intercellular CO_2_ concentration by 12.7% on average in DFG, but no significant effect or a slight increase was observed in HHBT, as indicated by a significant O_3_ × cultivar interaction (**Table 1**). This suggests that stomatal limitations play a key role in the decline of photosynthesis in DFG, while both stomatal and non-stomatal factors contribute to the decrease in HHBT. Moreover, significant O_3_-induced decreases in leaf *ETR* were identified, which may represent another non-stomatal mechanism of photosynthetic inhibition (Feng et al., 2016; Schcidegger and Schroeter, 1995).

Chlorophyll, a crucial component in leaf photosynthesis, plays a vital role in absorbing light, transferring energy, and converting it into chemical forms. Fluctuations in chlorophyll levels significantly influence nitrogen fixation and are indicative of leaf senescence (Singh et al., 2015). Consistent with the observed photosynthetic rate patterns, NF60 treatment led to reductions in chlorophyll a+b and its components in peony leaves, with chlorophyll a showing a more considerable decrease than chlorophyll b. While the high O_3_ concentration affected DFG’s chlorophyll content to a lesser extent than HHBT, no significant interaction was found between cultivar and O_3_ exposure. The changes in chlorophyll content under O_3_ stress likely reflect an imbalance in the production and scavenging of reactive oxygen species (ROS). Specifically, O_3_ exposure intensified lipid peroxidation in membranes, increasing MDA and H_2_O_2_ concentrations (**Table 4**), and allowing excess ROS to infiltrate chloroplasts, where they contributed to the degradation of chlorophyll. This is primarily due to the fact that these excess ROS exacerbate the peroxidation of chloroplast membranes, impairing the synthesis of chlorophyll-protein complexes (Langebartels et al., 2002). This process accelerates chlorophyll depletion and leaf yellowing, as extensively documented in crops such as rice (He et al., 2024) and wheat (Feng et al., 2016).

Leaf morphology and structure, shaped by the evolutionary processes of natural selection, are closely associated with physiological functions and represent key factors in determining a plant’s ability to adapt to its environment (Liu and Liang, 2016). The LMA is recognized as a reflection of plant adaptations and acclimation to prevailing environmental conditions. Species with low LMA display greater responsiveness to environmental stimuli and higher stress sensitivity compared to high-LMA species, which demonstrate enhanced stress tolerance (Bussotti, 2008; Li et al., 2016). The findings of this study indicated that NF60 exposure reduced leaf LMA by 12-13% (*P* < 0.1) in both peony cultivars, without any significant interaction observed between O_3_ exposure and cultivar, which aligns with similar reports in peach trees (Dai et al., 2017), maize (Peng et al., 2019), and rice (Fu et al., 2021). Stomatal traits, including density and size, are known to be highly responsive to environmental variations (Zheng and Shangguan, 2005). Previous research has reported differing responses of stomatal density to elevated O_3_, including increases (Wen et al., 2014), decreases (Li P. et al., 2018), or no significant effects (Xu et al., 2008b), which may be attributed to differences in exposure duration, O_3_ concentration, plant species, and the developmental stage of leaves (Li P, et al., 2018). Furthermore, no significant effects on stomatal density or size were found in peony leaves under O_3_ exposure (**Table 3**). This may be because the leaves were fully expanded before treatment, suggesting that the stomatal structure and number had already been established, thus minimizing the potential impact of the O_3_ exposure. Therefore, further long-term studies are needed to determine the effects of increased O_3_ on stomatal development throughout the lifespan of peony leaves.

The leaf antioxidant system plays a crucial role in defending against and repairing the oxidative damage caused by O_3_ stress. However, when environmental stress exceeds a critical threshold, it disrupts the plant’s protective defense mechanisms (Xu et al., 2020). Several studies have shown that O_3_-resistant species or cultivars typically display higher levels of antioxidant substances (Dai et al., 2017). In this study, although most leaf antioxidant parameters (such as AsA, TAC, total phenols, and the activities of enzymes like SOD, POD, and CAT) did not show statistically significant responses to O_3_ stress (**Table 4**), peony leaves treated with NF60 showed partial positive responses from their antioxidant system. This was accompanied by an increase in H_2_O_2_ and MDA content, with all antioxidant enzyme activities showing an upward trend. The results were largely consistent with the findings reported by Qin et al. (2020) regarding 80 ppb O_3_ stress on *Cleome* sp*inose*. Antioxidant indicators generally undergo dynamic changes, starting with an initial increase followed by a decrease as stress intensity increases (Wu et al., 2011). Therefore, measurements taken at a single time point may not adequately reflect the complete dynamic response. Future research should conduct multi-year experiments to elucidate the temporal variations of antioxidants in the cellular repair mechanisms of peony leaves.

## 5 Conclusions

Research on the effects of abiotic stress in peony has predominantly focused on factors such as temperature, light, water availability, salinity, and heavy metals. However, there have been no studies on the impact of atmospheric O_3_ pollution on peony growth. This study used open-top chambers to evaluate the effects of O_3_ stress on the leaf morphology and physiology of herbaceous peony. Compared with NF, the NF60 treatment led to a significant reduction in leaf photosynthetic capacity, as reflected by decreases in chlorophyll content and increases in MDA content, which contributed to accelerated leaf senescence in both peony cultivars tested. Under the same O_3_ exposure conditions, the two peony cultivars showed relatively minor changes in leaf morphology and physiology compared to crops such as soybean, rice, maize, and wheat, classifying peony as a species moderately sensitive to O_3_. Future research should focus on how elevated O_3_ concentrations affect medicinal compounds and key quality traits in cut flowers, such as stem length, flower color, and size. This knowledge will improve the comprehension of the physiological adaptation of peony to O_3_ stress, which could inform agronomic practices that optimize peony cultivation and commercialization and improve stress tolerance.

## Data Availability

The original contributions presented in the study are included in the article, further inquiries can be directed to the corresponding author/s.
